# Successful partial pulpotomy of a permanent mandibular molar: a two-year case report

**DOI:** 10.1038/s41415-025-8334-y

**Published:** 2025-03-14

**Authors:** Rose M. Thomas, Daniel Sisson

**Affiliations:** School of Medicine and Dentistry, University of Central Lancashire, Preston, PR1 2HE, UK

## Abstract

Emerging evidence reveals vital pulp therapy to be an alternative treatment modality for teeth that may have been considered candidates for root canal treatment or extraction. The aim of the procedure is to maintain the vitality of the tooth thus providing minimally invasive treatment. The procedure can be implemented in appropriate cases following clinical and radiographic examination. Direct examination of the exposed pulp tissue under magnification and strict aseptic conditions have been recommended for a successful outcome. This case report describes management of a patient with a deep caries lesion, involving the mesial pulp horn, using a vital pulp therapy procedure. The treatment options and discussions with the patient are highlighted together with the stages of the procedure carried out. A successful outcome of the partial pulpotomy is evidenced by clinical and radiographic examination two years post-treatment.

## Key points

- Vital pulp therapy procedures can be considered as an alternative to root canal treatment in appropriate cases.
- The aim of the procedure is to provide optimal conditions for repair to preserve the vitality of the tooth.
- This procedure is technically simple, affordable and effective.
- This case report describes successful management of a deep carious lesion using partial pulpotomy.

## Introduction

Minimally invasive techniques and preservation of healthy pulp are some of the key topics in contemporary clinical endodontics.^1^ Emerging evidence is pointing to the feasibility of vital pulp therapy (VPT) procedures for teeth that may have been deemed to have extraction or root canal treatment (RCT) previously.^2^

The rationale for VPT is based on the healing potential of the remaining pulp tissue and the aim of the procedure is to provide optimal conditions for repair in order to preserve the vitality of the tooth.^1^ The range of VPT procedures includes indirect pulp capping, direct pulp capping, partial pulpotomy and full pulpotomy.^1^ Historically, calcium hydroxide cement was the material of choice. However, there has been emerging evidence supporting use of bioceramic materials, such as mineral trioxide aggregate (MTA), to provide predictable results compared to calcium hydroxide.^3^^,^^4^^,^^5^^,^^6^ A recent systematic review of randomised clinical trials by Silva *et al*.^6^ concluded that pulpotomy is a highly effective treatment in the management of permanent teeth.

Although there is evidence pointing to successful VPT procedures, clinicians are hesitant to adopt this technique in day-to-day practice. This may be due to the lack of experience in doing this treatment, or ignorance about the emerging evidence. It is a valuable treatment modality recommendable to general dental practitioners as they are the primary point of contact for patients with pain, and before the tooth is extirpated, the VPT procedures can be implemented in appropriate cases.

Root-filled teeth have been reported to expedite tooth loss compared to non-root filled teeth.^7^^,^^8^ VPT in appropriate cases helps to retain the vitality of a tooth, preventing RCT and subsequent removal of hard tissue causing weakening of tooth structure,^9^ which in effect increase the longevity of the tooth. Although extended follow-up is recommended for long-term outcome assessment, VPT is considered a technically easier procedure compared to RCT, with reduced chair time and cost implication.^1^^,^^10^^,^^11^ This case report documents a successful treatment outcome of partial pulpotomy in a mandibular first molar with a deep carious lesion and with radiographic evidence of periapical changes.

## Case report

A 15-year-old female patient presented in a general dental practice with a deep buccal carious lesion on her left mandibular first permanent molar. The tooth was asymptomatic on presentation but had a history of mild-to-moderate pain a few weeks prior, for which she had been to the emergency dentist who placed a temporary filling which alleviated her symptoms. Clinically, there was some evidence of a filling material on the occlusal surface of the tooth.

The patient was a non-smoker, had a medical history of a heart murmur and was not on any medication other than contraceptive pills. Extraoral examination revealed no facial asymmetry or swelling and intraoral examination revealed no swelling or sinus tracts associated with the involved tooth. The teeth in the upper and lower arches were well-aligned with no crowding and presented with half unit Class II occlusion on both sides.

The tooth (left mandibular first permanent molar) demonstrated no tenderness to percussion or palpation, and pulp sensibility testing (Roeko Endo-Frost) demonstrated hypersensitivity to cold compared to the adjacent teeth, which did not last once the stimulus was removed. There was no lingering pain following the removal of the stimulus. Mobility was within normal limits and there was no clinical evidence of pocketing more than 3 mm around the tooth. The oral hygiene was also noted to be good. A diagnosis of reversible pulpitis was made based on the clinical tests. The diagnosis would be further confirmed while exploring the pulp clinically, assessing the health of the pulp and haemorrhage control.^12^

**Fig. 1:**
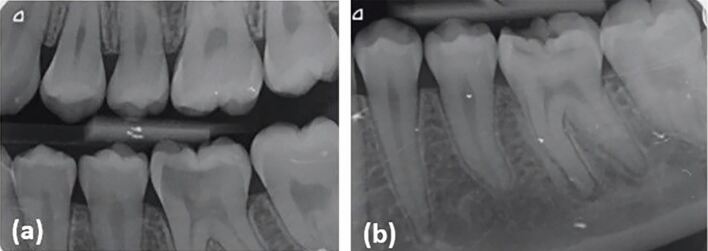
a) Pre-operative intraoral bitewing radiograph revealed extensive caries on the 36 involving the mesial pulp horn. b) Pre-operative periapical radiograph revealed evidence of apical radiolucency associated with the mesial root and periodontal ligament space widening of the distal root

Radiographic examination included an intraoral bitewing radiograph and a periapical radiograph (Fig. 1). There was evidence of periapical changes associated with the involved tooth and due to the increasing evidence emerging on the feasibility of VPT procedures in cases with periapical changes,^13^ the option to attempt VPT was discussed with the patient and parent.

Based on the clinical assessment and discussion with the patient, a shared decision was made to treat the tooth with a view to carry out VPT, depending on the health of the pulp tissue upon access. The patient and the parent were aware that if VPT was not indicated, then an RCT would be initiated on the visit.

**Fig. 2:**
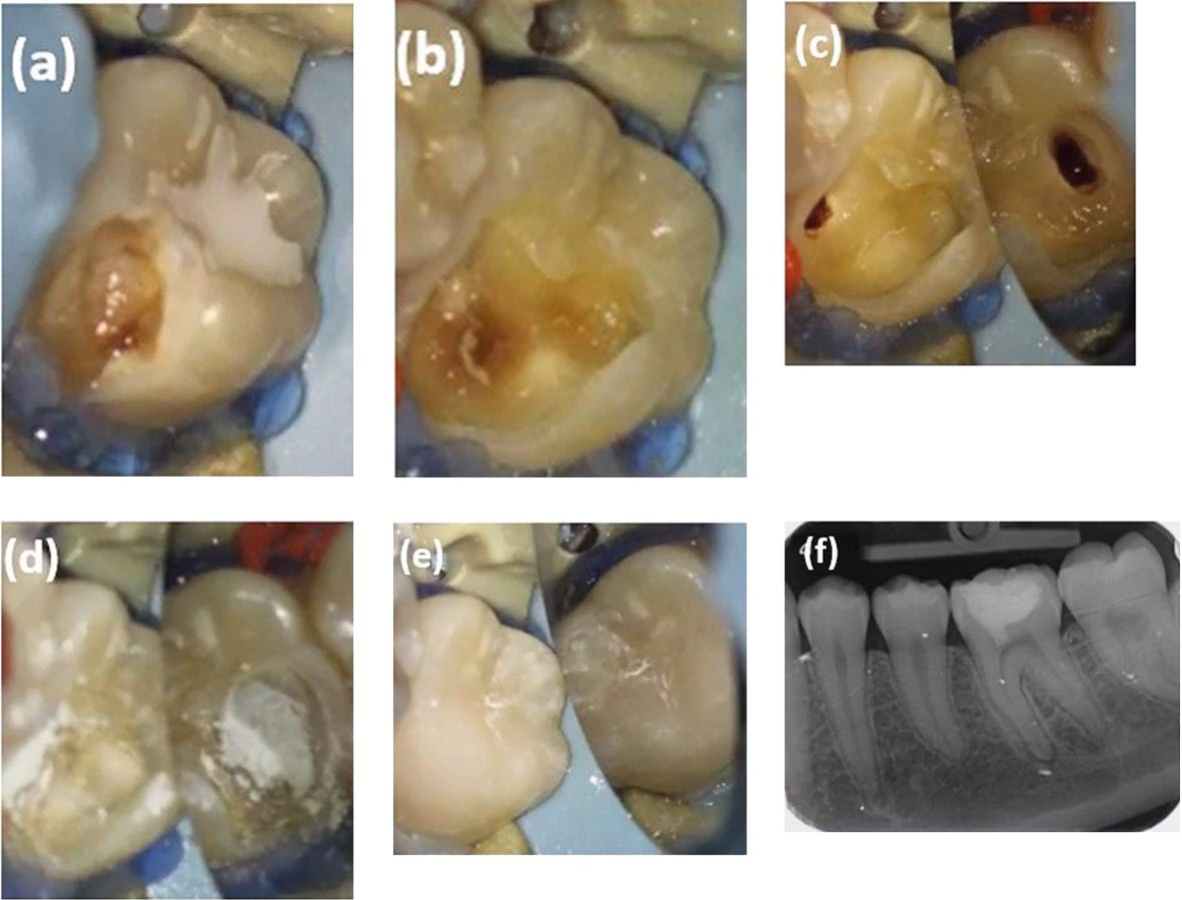
Clinical intraoral photographs. a) Pre-operative. b) Intraoperative - caries removal. c) Partial pulpotomy with haemorrhage control. d) MTA placement. e) Composite restoration. f) Post-operative periapical radiograph at baseline

Under local anaesthesia using 2% Xylocaine with 1:80,000 adrenaline and rubber dam isolation, the caries and remaining infected dentine were removed under a dental operating microscope (Carl Zeiss) using a high-speed handpiece and a sterile diamond bur with water coolant. Complete removal of the infected and soft dentine was ensured using a rose head bur in a slow handpiece. It was evident that the mesial pulp horn was involved and there was profuse bleeding from the exposure site. Coronal pulp tissue at the mesial pulp horn was removed using a sterile sharp spoon excavator. Haemostasis was achieved with the help of small sterile foam pellets soaked in 3% sodium hypochlorite (Calasept, Directa) solution for 2-3 minutes. Subsequent examination of the pulpotomy site under the operating microscope revealed healthy pulp tissue with blood clot (Fig. 2c). Freshly mixed MTA (Angelus White) was placed on the exposed pulp tissue and cavity (Fig. 2d) following the manufacturer's instruction, with the help of plastic instruments and burnisher. Once the MTA was set in 15 minutes, Riva light-cured resin-reinforced glass ionomer restorative cement (SDI) was used as a base, followed by permanent restoration with composite resin (Fig. 2e).

**Fig. 3:**
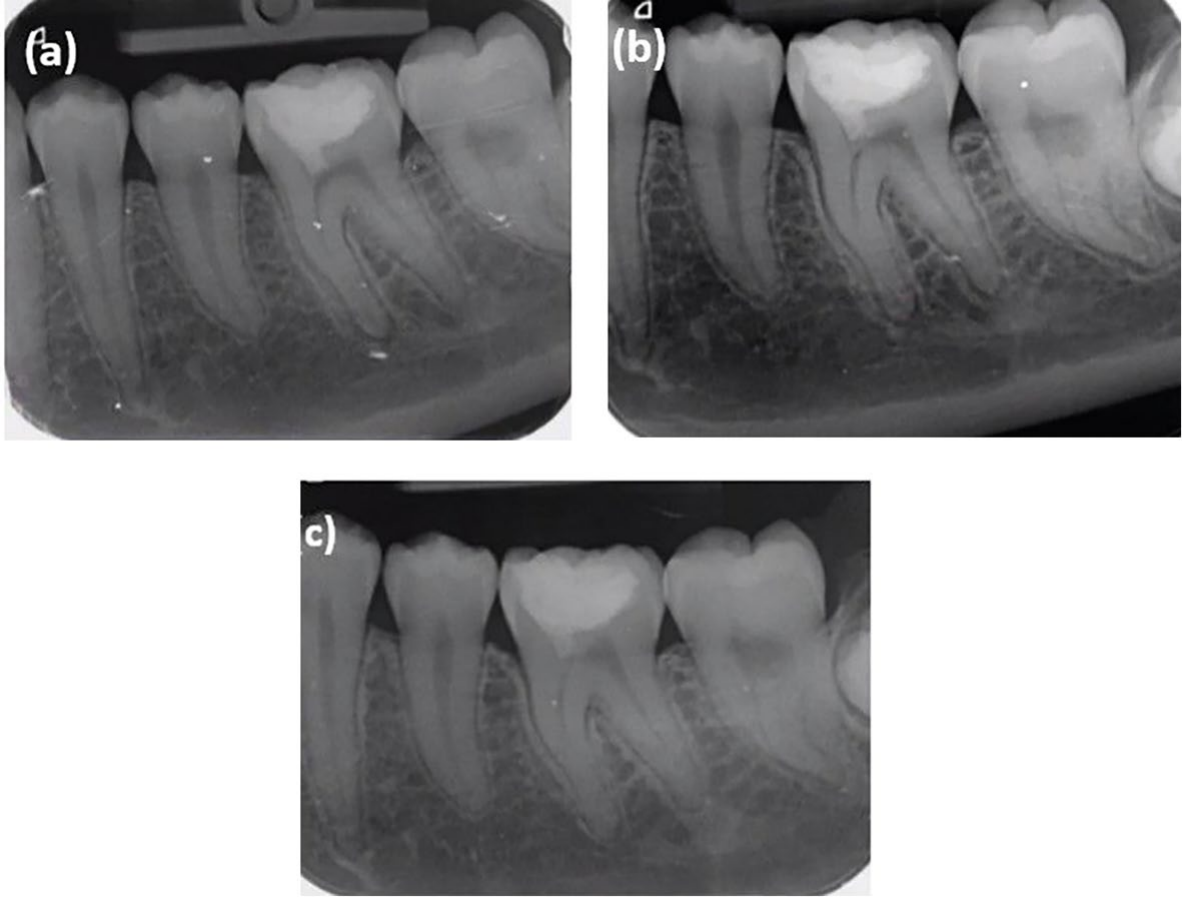
Post-operative radiographs. a) At baseline. b) At six months review. c) At two years and two months review

A post-operative intraoral periapical radiograph was taken at baseline (Fig. 2f, Fig. 3a) on the same visit, which revealed MTA placement in the mesial pulp horn.

A follow-up appointment was scheduled after six months for radiographic and clinical examination. On the follow-up visit, the patient did not report any pain or discomfort. The tooth was clinically asymptomatic and functional, and the periapical radiograph (Fig. 3b) revealed minor reduction in periapical changes associated with the mesial root.

**Fig. 4:**
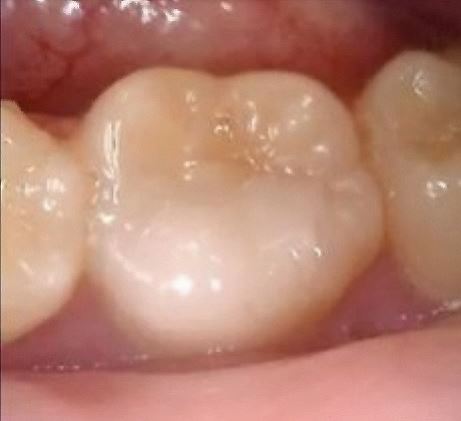
Clinical photograph of mandibular left first molar at two years and two months review appointment

A further follow-up was arranged for a year's time. However, as the patient re-arranged appointments, the next review was two years and two months after the treatment visit. The tooth was asymptomatic and functional on presentation. Clinical examination revealed pulpal response to a pulp sensibility test using Endo-Frost to be within normal limits and similar to the adjacent teeth. The tooth was not tender to percussion or palpation. There was no pathological mobility and there was no pocketing more than 3 mm around the tooth. An intraoral periapical radiograph was taken (Fig. 3c), which revealed good periapical healing, indicating that the treatment has been successful. No coronal discoloration was noted, the composite restoration was intact (Fig. 4) and the tooth was functional. However, further long-term follow-up was recommended to keep the tooth under monitoring, with clinical and radiographic evaluation, to diagnose asymptomatic pulp disease which may develop in the future.^14^

Although there was no clinical discoloration after two years in this case, there is evidence that bismuth oxide-containing cements, such as MTA, can cause tooth discoloration, especially with the use of sodium hypochlorite.^15^ Hence, its use should be avoided in anterior teeth or aesthetically important teeth. Other bioceramic materials, such as Biodentine, which does not contain bismuth oxide, could be used in such situations.

## Discussion

RCT is one of the most technique-sensitive clinical procedures, especially in multi-rooted teeth.^10^ To provide a good-quality RCT, the clinician needs to have considerable experience, advanced equipment, and materials, as well as extensive clinical time. With the advent of bioceramic materials, it is now possible to have VPT as an alternative conservative treatment in appropriate cases, which is technically simple, affordable and effective.^10^

Traditionally, it was thought that there is poor co-relation between clinical symptoms of pulpitis and the histological state of a diseased pulp, but a histological study by Ricucci *et al*.^16^ proved otherwise. The study also found that histological analysis of cases with irreversible pulpitis revealed viable radicular pulp, with areas of coagulation or liquefaction necrosis principally in the coronal pulp. The difficulty in predictably assessing the potential degree of inflammation of the pulp was addressed by Wolters *et al*.,^17^ who recommended a new diagnostic system for assessing pulpitis and subsequent treatment need.

VPT procedures include distinct treatment modalities and the indication and rationale can be confusing to the general dental practitioner. A histopathological, histobacterial and clinical findings-based study proposed recommendations and guidelines for VPT, which involve direct examination of the dentine and the exposed pulp tissue under strict aseptic conditions.^18^ There is a growing body of evidence revealing successful outcome with VPT, even in cases with signs and symptoms of irreversible pulpitis with or without apical lesions.^13^^,^^19^^,^^20^^,^^21^ A randomised clinical trial comparing partial pulpotomy and complete pulpotomy on mature permanent teeth with symptomatic irreversible pulpitis found favourable outcomes with both partial and complete pulpotomy.^11^ They recommended attempting partial pulpotomy first due to the conservative nature of the procedure.

An important finding in this case report is the complete healing of the periapical lesion (Fig. 3c) without any further treatment need. This tooth would have been a candidate for RCT and a cuspal coverage restoration if the VPT was not attempted. This would have resulted in undue weakening of this tooth due to removal of hard tissue for RCT, as well as preparation for cuspal coverage restoration, which in turn will result in reducing the longevity of the tooth itself. The more conservative the restorative treatment, the more chance of preserving the pulp vitality.^12^ The clinician must choose restorative options, whether that be cuspal coverage restoration or direct adhesive restoration, which could provide a predictable seal against bacterial leakage, depending on the individual assessment of the tooth.

Changes in clinical practice should be recommended by governing bodies, which could benefit the population in the long-run, reducing dental treatment needs. The current NHS (National Health Service) band systems with units of dental activity have resulted in an exodus of dentists from the NHS. It has been reported that this band system has made dentists to feel that complex treatments are financially unviable for their business.^22^ VPT, which is not as complex as RCT, is an affordable alternative treatment in general dental practice. This treatment modality has delivered clinical outcomes similar to that of conventional RCT.^23^ Dentists should be encouraged to attempt to preserve vitality of teeth, thus providing evidence-based, minimally invasive dentistry, so that the general public can retain their teeth with minimal maintenance.

## Conclusion

This case report illustrates a successful treatment outcome of partial pulpotomy in a tooth with pulpal and periapical involvement. This procedure has helped to retain the vitality of this tooth, thus avoiding technique-sensitive RCT, as well as the need for cuspal coverage restoration, which could affect the longevity of the tooth itself. This case report provides evidence to clinical practice that partial pulpotomy procedures can be considered as an alternative procedure to RCT in appropriately selected cases.
